# Progression of motor subtypes in Huntington’s disease: a 6-year follow-up study

**DOI:** 10.1007/s00415-016-8233-x

**Published:** 2016-07-19

**Authors:** M. Jacobs, E. P. Hart, E. W. van Zwet, A. R. Bentivoglio, J. M. Burgunder, D. Craufurd, R. Reilmann, C. Saft, R. A. C. Roos, The REGISTRY investigators of the European Huntington’s Disease Network

**Affiliations:** 1 Department of Neurology, Leiden University Medical Center, Albinusdreef 2, 2333 ZA Leiden, The Netherlands; 2 Center for Human Drug Research, Leiden, The Netherlands; 3 Department of Medical Statistics, Leiden University Medical Center, Leiden, The Netherlands; 4 Department of Neurology, Università Cattolica del Sacro Cuore, Rome, Italy; 5 Fondazione Don Carlo Gnocchi, Milan, Italy; 6 Department of Neurology, University of Bern, Bern, Switzerland; 7 Faculty of Medical and Human Sciences, Institute of Human Development, University of Manchester, Manchester, UK; 8 Manchester Center of Genomic Medicine, St. Mary’s Hospital, Central Manchester University Hospitals NHS Foundation Trust and Manchester Academic Health Science Center, Manchester, UK; 9 George Huntington Institute, Muenster, Germany; 10 Department of Radiology, University of Muenster, Muenster, Germany; 11 Department of Neurodegenerative Diseases and Hertie-Institute for Clinical Brain Research, University of Tuebingen, Tuebingen, Germany; 12 Department of Neurology, Ruhr-University Bochum, Bochum, Germany

**Keywords:** Huntington’s disease, Chorea, Hypokinetic, Neuropsychological assessment

## Abstract

The objective of this study is to investigate the progression of predominantly choreatic and hypokinetic-rigid signs in Huntington’s disease (HD) and their relationship with cognitive and general functioning over time. The motor signs in HD can be divided into predominantly choreatic and hypokinetic-rigid subtypes. It has been reported in cross-sectional studies that predominantly choreatic HD patients perform better on functional and cognitive assessments compared to predominantly hypokinetic-rigid HD patients. The course of these motor subtypes and their clinical profiles has not been investigated longitudinally. A total of 4135 subjects who participated in the European HD Network REGISTRY study were included and classified at baseline as either predominantly choreatic (*n* = 891), hypokinetic-rigid (*n* = 916), or mixed-motor (*n* = 2328), based on a previously used method. The maximum follow-up period was 6 years. The mixed-motor group was not included in the analyses. Linear mixed models were constructed to investigate changes in motor subtypes over time and their relationship with cognitive and functional decline. Over the 6-year follow-up period, the predominantly choreatic group showed a significant decrease in chorea, while hypokinetic-rigid symptoms slightly increased in the hypokinetic-rigid group. On the Total Functional Capacity, Stroop test, and Verbal fluency task the rate of change over time was significantly faster in the predominantly choreatic group, while on all other clinical assessments the decline was comparable for both groups. Our results suggest that choreatic symptoms decrease over time, whereas hypokinetic-rigid symptoms slightly increase in a large cohort of HD patients. Moreover, different motor subtypes can be related to different clinical profiles.

**Electronic supplementary material:**

The online version of this article (doi:10.1007/s00415-016-8233-x) contains supplementary material, which is available to authorized users.

## Introduction

Huntington’s disease (HD) is an autosomal dominant neurodegenerative disorder characterized by motor disturbances, cognitive dysfunction and psychiatric symptoms [1, 2]. When the disease progresses, motor impairments increase and patients become more impaired in their daily-life activities [2]. The primary motor disturbances in HD are generally categorized as involuntary choreatic movements [2]. However, hypokinesia is also a core component of HD [3–5], and cannot solely be attributed to medication usage [4]. It has been reported that chorea is often more pronounced in patients with early stage HD, whereas in advanced stages of HD hypokinetic symptoms become more dominant [6–8]. However, the clinical motor phenotype is heterogeneous and chorea and hypokinesia can also co-exist [9].

Another clinical feature of HD is the decline in cognitive functioning, especially in the executive domain [10, 11]. These cognitive deficits can be present years before motor signs become overt [12]. To date, a growing number of studies have focused on the relationship between motor, functional and cognitive impairments [4, 13–17]. Recently, a cross-sectional study showed that predominantly choreatic HD patients perform better on general and cognitive assessments compared to predominantly hypokinetic-rigid HD patients, and that this cannot be explained by differences in age or disease duration [13]. This suggests that chorea is associated with less cognitive and global impairment than hypokinesia. However, the course of motor symptoms has not been studied longitudinally. Therefore, we investigated the progression of motor symptoms over time in different motor subtypes and examined their relationship with cognitive and functional decline to get a better understanding of the clinical course.

## Methods

Data of subjects with a confirmed Cytosine-Adenine-Guanine (CAG) repeat length of ≥36 on the larger allele and a total motor score (TMS) of >5 on the Unified HD Rating Scale (UHDRS) [18] at baseline, who participated in the REGISTRY study of the European HD Network (EHDN) were used for this study. The local medical ethics committee approved the study and written informed consent was obtained from all participants.

All subjects completed at least one follow-up visit, with the TMS rated five or higher during all follow-up visits, to ensure the motor presentation of HD. The maximum follow-up period in this study was 6 years. Subjects with a disease onset before the age of 21 were considered juvenile HD and were excluded from the analyses. A total of 4135 subjects fulfilled the criteria for this study. The classification into motor subtypes at baseline was based on the method used in our previous cross-sectional study [13]. Total chorea and total hypokinetic-rigid scores were calculated by adding items of the UHDRS, with a maximum of 28 for each score. For the hypokinetic-rigid score, items 6, 7, 9, and 10 of the UHDRS were added. Item 12 of the UHDRS was used to calculate the choreatic score. The difference between the two total scores had to be greater than one standard deviation (i.e. 4 points) to divide the predominantly subtypes. A subject was considered mixed-motor if the difference was smaller than one standard deviation. This classification resulted in 891 predominantly choreatic, 916 predominantly hypokinetic-rigid, and 2328 mixed-motor subjects at baseline. Disease burden was calculated using the formula (age * (CAG repeat length − 35.5)) [19]. Subjects were categorized into disease stages (1–5) based on the UHDRS total functional capacity (TFC) score, which is a measure to assess general functioning (range 0–13) [20]. The cognitive battery consisted of the written Symbol Digit Modalities test (SDMT), Stroop test (color, word, and interference), and Verbal fluency task. Of the 4135 subjects, a total of 2446 subjects (436 predominantly hypokinetic-rigid, 554 predominantly choreatic, 1456 mixed-motor) completed at least one cognitive task at baseline and at least one follow-up visit. These subjects were included in the analyses regarding the relationship between motor subtypes and clinical measures over time.

### Statistical analyses

IBM Statistical Package for the Social Sciences (SPSS) 20 for Windows was used for data analyses. Group comparisons at baseline were performed using parametric (independent sample *t* tests) and non-parametric tests (*χ*^2^ test and Mann–Whitney *U* test). The use of neuroleptics was scored as ‘0 = present’ or ‘1 = absent’. An overview of medication considered neuroleptics is reported in Supplementary Material 1.

A multilevel regression model (i.e., linear mixed model), adjusting for age at baseline, gender, CAG repeat length, and TMS was constructed to investigate the course of the different motor subtypes over time. Since we were primarily interested in the differences between the predominant motor subtypes, the mixed-motor group was not included in the analyses. Differences between total hypokinetic-rigid and total chorea scores (total chorea − total hypokinetic-rigid) from each time point were used as outcome variable. Negative scores indicated more hypokinetic-rigid symptoms, while positive scores indicated more choreatic symptoms. Time was measured in months since baseline. To account for the correlation between repeated measurements on the same subject, a random intercept and random time effect (slope) per subject was used. To investigate the relationship between motor subtypes and clinical measures, separate linear mixed models, adjusting for age at baseline, gender, CAG repeat length, TMS, and years of education were constructed. Total scores per cognitive assessment and the TFC were used as outcome variables. For all analyses an unstructured covariance for the random intercepts and random slopes was used. Differences at baseline and rate of change (i.e., slope) between both motor groups were compared.

## Results

Demographic data at baseline are shown in Table 1. The predominantly hypokinetic-rigid group was significantly younger, more often female, had higher CAG repeat lengths, a longer disease duration, higher disease burden scores, lower TFC scores, and a higher TMS at baseline compared to the choreatic group. There was no difference between the two groups in number of subjects using neuroleptics.

**Table 1:** Demographics of whole group and separate motor groups at baseline

	Whole group	Hypokinetic-rigid	Choreatic	*p* value hypokinetic-rigid vs choreatic
*N*	4135	916	891	
Age, years	51.8 (11.8)	51.8 (12.5)	53.1 (11.6)	0.025
Gender, m/f (%m)	1998/2137 (48.3)	419/497 (45.7)	479/412 (53.8)	<0.005
Neuroleptics, y/n (%y)	3393/742 (82.1)	762/154 (83.2)	743/148 (83.4)	0.909
CAG repeat length	44.0 (3.4)	44.8 (3.8)	43.5 (3.2)	<0.001
Disease duration, years	6.4 (5.3)	7.8 (5.9)	6.1 (4.9)	<0.001
Disease burden	409.1 (107.2)	440.2 (118.6)	394.7 (102.9)	<0.001
UHDRS TMS	35.9 (20.0)	47.2 (22.1)	34.7 (17.2)	<0.001
UHDRS TFC	9.0 (0–13)	5.0 (0–13)	10.0 (0–13)	<0.001
HD stage (%)	*N* = 4005	*N* = 887	*N* = 864	
1	1382 (34.5)	120 (13.5)	373 (43.2)	
2	1309 (32.7)	223 (25.1)	290 (33.6)	
3	954 (23.8)	305 (34.4)	169 (19.6)	
4	293 (7.3)	190 (21.4)	29 (3.4)	
5	67 (1.7)	49 (5.5)	3 (0.3)	

Data are mean (SD) for continuous variables, median (range) for UHDRS TFC, and number (%) for gender, neuroleptics, and HD stage. Analyses are independent sample *t* tests, except for UHDRS TFC (Mann–Whitney *U* test), gender and neuroleptics (*χ* ^2^ test). Number (%) of HD stages are based on different sample sizes due to missing data

*CAG* Cytosine-Adenine-Guanine, *UHDRS TMS* Unified Huntington’s Disease Rating Scale Total Motor Score, *UHDRS* *TFC* Unified Huntington’s Disease Rating Scale Total Functional Capacity, *HD* Huntington’s disease

**Fig. 1:**
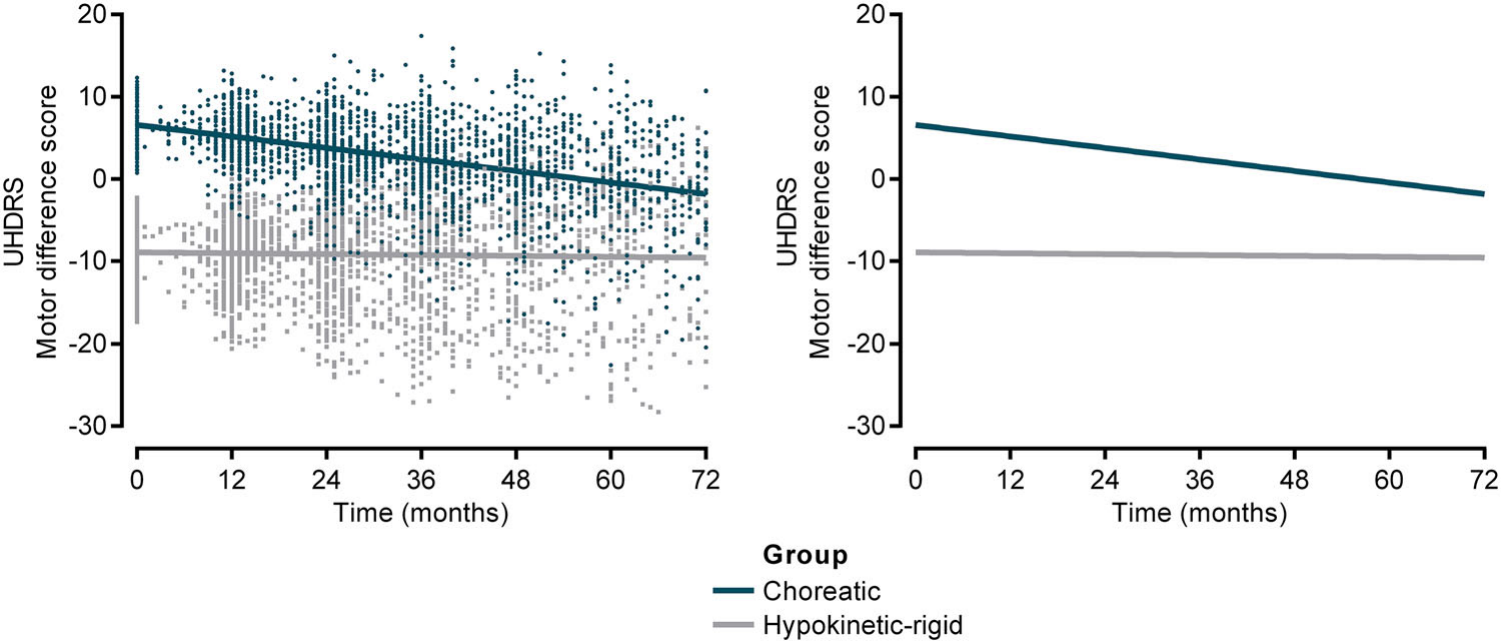
Predicted values (*left*) and fitted longitudinal curves (*right*) of the motor difference score for each motor subgroup. Predicted values and longitudinal curves are based on the linear mixed model. *UHDRS* Unified Huntington’s Disease Rating Scale

The linear mixed model, adjusted for age at baseline, gender, CAG repeat length, and TMS showed significantly lower baseline scores for the predominantly hypokinetic-rigid group on the motor difference score (Table 2). The predominantly choreatic group showed a more rapid decline on the motor difference score over the 6-year follow-up period compared to the hypokinetic-rigid group (*β* = 0.11, SE = 0.01, *p* value <0.001) (Fig. 1). There was a significant effect of time for both groups (*β* = −0.12, SE = 0.01, *p* value <0.001).

**Table 2:** Baseline and slope differences for the hypokinetic-rigid vs choreatic group

	Hypokinetic-rigid vs choreatic	*p* value
Motor course		
Baseline difference	−14.95 (0.21)**	<0.001
Slope difference	0.11 (0.01)**	<0.001
TFC score		
Baseline difference	−2.31 (0.14)**	<0.001
Slope difference	0.02 (0.00)**	<0.001
SDMT		
Baseline difference	−4.44 (0.65)**	<0.001
Slope difference	0.02 (0.01)	0.100
Stroop color reading		
Baseline difference	−6.86 (0.83)**	<0.001
Slope difference	0.03 (0.02)	0.098
Stroop word reading		
Baseline difference	−11.11 (1.12)**	<0.001
Slope difference	0.08 (0.03)*	0.003
Stroop interference		
Baseline difference	−4.64 (0.59)**	<0.001
Slope difference	0.02 (0.02)	0.137
Verbal fluency		
Baseline difference	−3.98 (0.70)**	<0.001
Slope difference	0.03 (0.01)*	0.021

Shown are parameter estimates (SE) from the linear mixed models

*TFC* total functional capacity, *SDMT* Symbol Digit Modalities test

* *p* < 0.05, ** *p* < 0.001 indicate significant differences in the hypokinetic-rigid group compared to the choreatic group

**Fig. 2:**
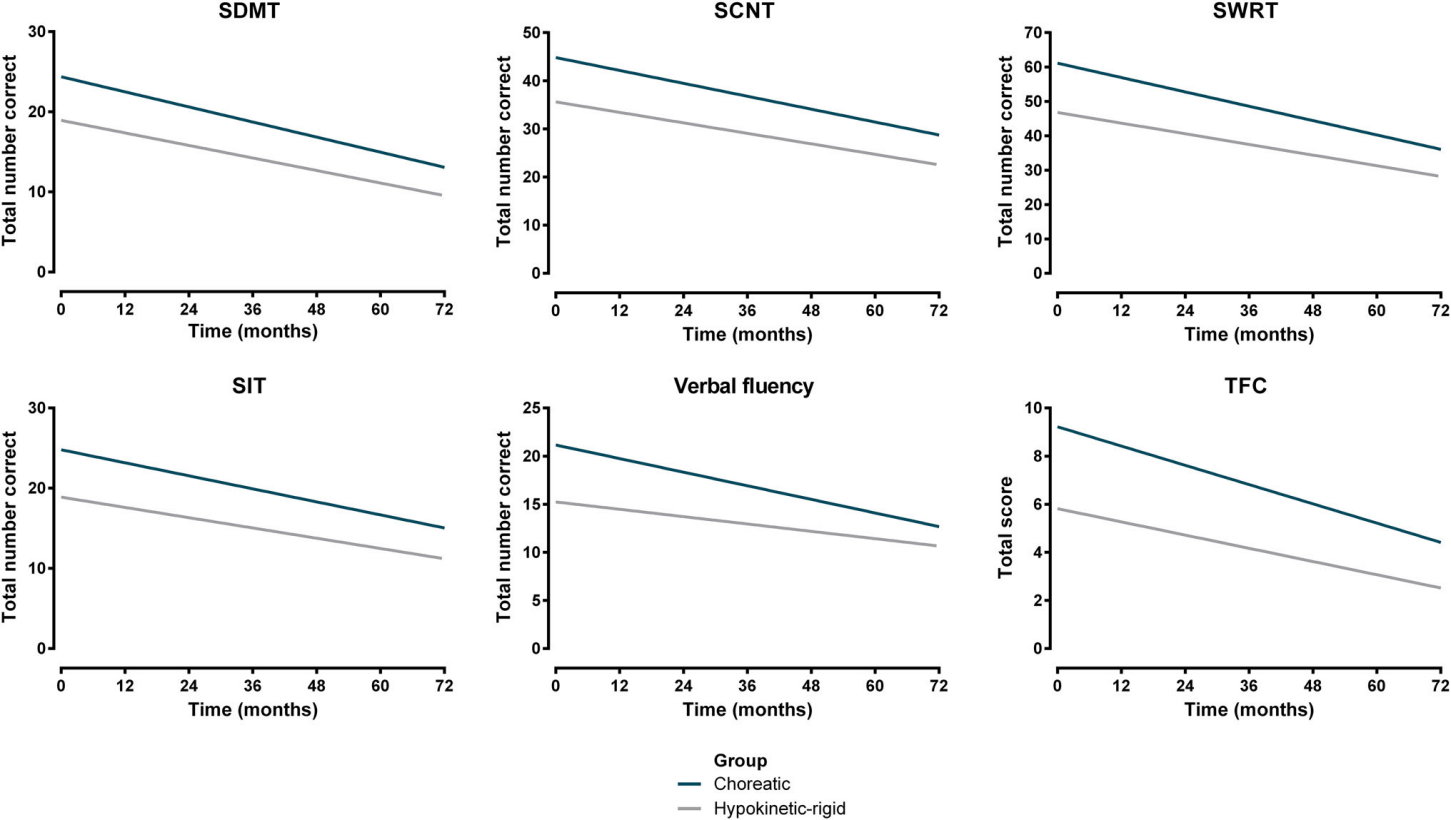
Fitted longitudinal curves for all clinical assessments for each motor subgroup. *SDMT* Symbol Digit Modalities test, *SCNT* Stroop color naming test, *SWRT* Stroop word reading test, *SIT* Stroop interference test, *TFC* total functional capacity

Linear mixed models adjusted for age at baseline, gender, CAG repeat length, TMS, and years of education showed lower baseline scores for the predominantly hypokinetic-rigid group compared to the predominantly choreatic group on all clinical measures (Table 2). For all assessments there was a significant effect of time, with both groups deteriorating (data not reported in Table 2). Significant differences in change over time between the two groups were observed for the TFC, Stroop word reading (SWRT), and Verbal fluency task, with the choreatic group showing a slightly faster rate of decline on these tasks (Table 2; Fig. 2). Overall, the predominantly choreatic group had better performances over time than the predominantly hypokinetic-rigid group on all clinical assessments (Fig. 2).

Secondary analyses performed in the non-medication group (*n* = 302) showed similar significant results for both motor subtypes on all outcome variables compared to the whole group analyses (data not reported).

## Discussion

This longitudinal study in a large cohort of HD patients showed that there is a significant difference in the progression of motor symptoms between predominantly hypokinetic-rigid and predominantly choreatic subjects over a 6-year follow-up period. Also, an association between motor subtypes and clinical assessments was observed.

The predominantly choreatic group showed a more rapid decline on the motor difference score compared to the hypokinetic-rigid group. This suggests that motor phenotype can be a predictor for a different progression of motor symptoms, with the predominantly choreatic group showing a decrease in chorea and the hypokinetic-rigid group a slight increase of hypokinetic-rigid signs during the course of 6 years. Thus, the progression of motor symptoms is not uniform for both subtypes. These results strengthen the ideas proposed in previous studies stating that the presence of chorea is often more pronounced in early HD stages, and slowly decreases with time [7, 8]. The observed differences cannot be explained by differences in age at baseline, since these were accounted for in the analyses. Moreover, since we observed an effect of time for both groups, our results do not seem to indicate a regression to the mean phenomenon. Our study is the first study to investigate the course of motor phenotypes longitudinally and to relate these phenotypes with cognitive and functional assessments. The differences between both groups at baseline for all cognitive and functional assessments indicates that predominantly choreatic subjects perform better on clinical assessments compared to predominantly hypokinetic-rigid subjects. This is consistent with the results reported in our previous cross-sectional study [13]. At baseline, there were significantly more females, lower TFC scores, and a higher UHDRS TMS observed in the predominantly hypokinetic-rigid group compared to the choreatic group. Gender has been associated with differences in disease progression and UHDRS TMS, with females showing poorer scores and faster progression rates compared to men [21]. These results might be related to the differences reported in our study. However, the longitudinal analyses in our study were adjusted for gender and TMS, so baseline differences between the groups cannot explain the different progression of motor symptoms that was observed between both subtypes. Future studies should be conducted to explore the influence of gender on motor subtype to increase the knowledge about differences in progression rate and phenotypes.

On the TFC, SWRT, and Verbal fluency task a significant difference in rate of change over time between the two groups was found, which implies that the course of cognitive deterioration can differ for each motor subtype. The predominantly choreatic group showed a slightly faster decline on these three tasks compared to the hypokinetic-rigid group. This suggests that the decline in cognitive and general functioning might be more rapid in this group compared to the hypokinetic-rigid group. These differences in rate of change over time could potentially be the result of the large study cohort and the linear design of the analyses, and should be interpreted with caution. However, it should be noted that on all other tasks the change over time between both groups was comparable. During the whole 6-year follow-up period, the predominantly choreatic group continued to perform better than the hypokinetic-rigid group on all clinical assessments. Although both groups showed a deterioration in cognitive and functional performances, the differences between the motor subtypes observed at baseline remained during the course of 6 years. These findings are consistent with previous studies reporting that hypokinesia is associated with cognitive and functional impairments [3, 4, 14, 15]. The lack of a relationship between chorea and cognitive functioning that is often reported [15, 17], might be explained by the fact that choreatic patients perform better on these assessments during the course of the disease. Thus, motor phenotype might be a predictor for differences in cognitive and functional profiles. This should be considered in the future development of clinical trials and in choosing the right clinical endpoints. Including only subjects with a particular motor phenotype could potentially affect the outcome of a clinical trial, since the performances of each phenotype might differ.

Some limitations of our study should be mentioned. Due to the longitudinal nature of this study it is likely that subjects who show the most rapid cognitive decline are lost to follow-up, which makes our study more prone to attrition bias. Additionally, the cognitive assessments we used can only provide information about certain cognitive domains. Including a more extensive cognitive battery might provide insights into other cognitive domains that are also affected in HD. Although there were no differences between the two motor subgroups in the use of neuroleptics at baseline, and secondary analyses in the non-medication group showed similar results for both subgroups compared to the whole group analyses, we did not investigate the influence of medication usage on the classification of motor subtypes. The REGISTRY study provides limited information about treatment dosage and duration, which complicates the analyses of medication usage. Future studies should be conducted investigating the relation between medication and motor phenotypes more thoroughly, since it might induce hypokinetic symptoms. This could potentially lead to a misclassification of subjects into the hypokinetic-rigid group. The mixed-motor group was the largest group in our study, which indicates that most patients will express both chorea and hypokinetic-rigid symptoms. Investigating the characteristics of this group could perhaps provide more insight into the changes in motor and clinical scores over time for an extensive amount of patients.

In conclusion, this longitudinal study found that choreatic symptoms decrease over time, whereas hypokinetic-rigid symptoms slightly increase in a large cohort of HD patients. Moreover, different motor subtypes can be related to different clinical profiles.

## Electronic supplementary material

Below is the link to the electronic supplementary material.

Supplementary material 1 (DOCX 19 kb)

Supplementary material 2 (DOC 93 kb)
