# Supplementary material for: Progression of motor subtypes in Huntington’s disease: a 6-year follow-up study
Source: J Neurol. 2016 Jul 19;263(10):2080–5. doi: 10.1007/s00415-016-8233-x (PMC5037142; doi:10.1007/s00415-016-8233-x)
Supplement: Supplementary file 1 — Supplementary material 1 (DOCX 19 kb) [file 415_2016_8233_MOESM1_ESM.docx]

**Supplementary Material 1 Overview of medications considered neuroleptics**

| **Medication type** | **Name of medication** |
| --- | --- |
| Antipsychotics | Olanzapine * |
|  | Tiapride * |
|  | Risperidal * |
|  | Sulpiride * |
|  | Haloperidol * |
|  | Quetiapine |
|  | Pimozide |
|  | Aripiprazole |
|  | Clozapine |
|  | Amisulpride |
|  | Ketrel |
|  | Pipamperon |
|  | Melperon |
|  | Levomepromazine |
|  | Promazine |
|  | Levosulpiride |
|  | Cyamemazine |
|  | Chlorprothixen |
| Antidepressants | Citalopram * |
|  | Mirtazapine * |
|  | Sertraline * |
|  | Escitalopram |
|  | Paroxetine |
|  | Venlafaxine |
|  | Fluoxetine |
|  | Amitriptyline |
|  | Clomipramine |
|  | Mianserine |
|  | Duloxetine |
|  | Doxepin |
|  | Bupropion |
|  | Fluvoxamine |
|  | Trimipramine |
|  | Lithium |
| Antiepileptics | Sodium valproate |
|  | Carbamazepine |
|  | Pregabaline |
|  | Lamotrigine |
|  | Neurontin |
|  | Depakine |
|  | Levetiracetam |
|  | Oxcarbazepine |
| Benzodiazepines | Clonazepam * |
|  | Lorazepam |
|  | Diazepam |
|  | Alprazolam |
|  | Zopiclon |
|  | Oxazepam |
|  | Zolpidem |
|  | Bromazepam |
|  | Piracetam |
|  | Lormetazepam |
|  | Temazepam |
|  | Tranxilium |
|  | Delorazepam |
|  | Nitrazepam |
|  | Tetrazepam |
|  | Flurazepam |
|  | Elenium |
| Opioids | Tramadol |
| Others | Tetrabenazine * |
|  | Memantine |
|  | Domperidon |
|  | Riluzole |
|  | Metoclopramide |
|  | Modafinil |
|  | Rivastigmine |
|  | Donepezil |
|  | Amantadine |
|  | Akineton |
|  | Madopar |
|  | Levodopa |
|  | Trihexylfenidyl |

* = 10 most frequently used medications in the cohort studied.
