# Supplementary material for: Progression of motor subtypes in Huntington’s disease: a 6-year follow-up study
Source: J Neurol. 2016 Jul 19;263(10):2080–5. doi: 10.1007/s00415-016-8233-x (PMC5037142; doi:10.1007/s00415-016-8233-x)
Supplement: Supplementary file 2 — Supplementary material 2 (DOC 93 kb) [file 415_2016_8233_MOESM2_ESM.doc]

**Supplementary Material 2 Acknowledgement list REGISTRY investigators**

REGISTRY 2004- 2015

**Registry Steering committee:** Anne-Catherine Bachoud-Lévi, Anna-Rita Bentivoglio, Ida Biunno, Raphael M. Bonelli, Juliana Bronzova, Jean-Marc Burgunder, Stephen B. Dunnett, Joaquim J. Ferreira, Jan Frich, Joe Giuliano, Olivia J. Handley, Arvid Heiberg, Sergey Illarioshkin, Torsten Illmann, Jiri Klempir, G. Bernhard Landwehrmeyer, Jamie Levey, Tim McLean, Jørgen E. Nielsen, Susana Pro Koivisto, Markku Päivärinta, Sven Pålhagen, Oliver Quarrell, Maria Ramos-Arroyo, Raymund A.C. Roos, Carsten Saft, Ana Rojo Sebastián, Sarah J. Tabrizi, Wim Vandenberghe, Christine Verellen-Dumoulin, Tereza Uhrova, Jan Wahlström+, Jacek Zaremba

**Language coordinators:** Verena Baake (formerly Rödig), Katrin Barth, Monica Bascuñana Garde, Tomáš Bernard, Sabrina Betz, Reineke Bos, Adrien Come, Leonor Correia Guedes, Jenny Callaghan, Selene Capodarca, Sébastien Charpentier Wildson, Vieira da Silva, Martina Di Renzo, Daniel Ecker, Ana Maria Finisterra,, Ruth Fullam, Camille Genoves, Mette Gilling, Olivia J Handley, Carina Hvalstedt, Christine Held, Hasina Hussain, Kerstin Koppers, Claudia Lamanna, Matilde Laurà, Asunción Martínez Descals, Saul Martinez-Horta, Tiago Mestre, Sara Minster, Daniela Monza, Kristina Münkel, Lisanne Mütze, Martin Oehmen, Helene Padieu, Laurent Paterski, Nadia Peppa, Susana Pro Koivisto, Beate Rindal, Dawn Rogers, Niini Røren (formerly Heinonen), Ana Salgueiro, Pavla Šašinková, Yury Seliverstov, Catherine Taylor, Erika Timewell, Jenny Townhill, Patricia Trigo Cubillo, Marleen R van Walsem, Marie-Noelle Witjes-Ané, Grzegorz Witkowski, Abigail Wright, Elizaveta Yudina, Daniel Zielonka, Eugeniusz Zielonka, Paola Zinzi

**AUSTRIA**

**Graz (Medizinische Universitäts Graz, Psychiatrie):** Raphael M. Bonelli, Karen Hecht, Brigitte Herranhof, Anna Holl (formerly Hödl), Hans-Peter Kapfhammer, Michael Koppitz, Sabine Lilek, Markus Magnet, Nicole Müller, Daniela Otti, Annamaria Painold, Karin Reisinger, Monika Scheibl, Helmut Schöggl, Jasmin Ullah

**Innsbruck (Universitätsklinik Innsbruck, Neurologie):** Eva-Maria Braunwarth, Florian Brugger, Lisa Buratti, Eva-Maria Hametner, Caroline Hepperger, Christiane Holas, Anna Hotter, Anna Hussl, Barbara Larcher, Philipp Mahlknecht, Christoph Müller, Bernadette Pinter, Werner Poewe, Eva-Magdalena Reiter, Klaus Seppi, Fabienne Sprenger, Gregor Wenning

**Salzburg (Christian-Doppler-Klinik Salzburg, Universitätsklinikum der PMU, Universitätsklinik für Neurologie):** Raphael M. Bonelli, Gunther Ladurner, Stefan Lilek, Daniela Sinadinosa, Wolfgang Staffen, Anna Maria Walleczek

**BELGIUM**

**Bierbeek:** Dirk Liessens, Godelinde Calmeyn, Nele Somers, Isabelle Delvaux, Andrea Boogaerts

**Bruxelles (Erasme):** Nick Alaerts, Frédéric Supiot

**Charleroi (Institut de Pathologie et de Génétique (IPG)):** Michel Dupuis, Cécile Minet, Pascale Ribaï, Dominique Van Paemel, Christine Verellen-Dumoulin

**Leuven:** (Universitair Ziekenhuis Gasthuisberg,): Andrea Boogaerts, Wim Vandenberghe, Dimphna van Reijen, Petra Weckx

**CZECH REPUBLIC**

**Prague (Extrapyramidové centrum, Neurologická klinika, 1. LF UK a VFN):** Jiří Klempíř, Veronika Majerová, Jan Roth

**DENMARK**

**Aarhus (Aarhus University Hospital):** Louise Hasselstrøm Madsen, Anette Torvin Møller

**Copenhagen University Hospital (Rigshospitalet, Memory clinic):** Lena Hjermind, Oda Jacobsen, Suzanne Lindquist, Jørgen Nielsen, Lisbeth Regeur, Jette Stockholm, Ida Unmack Larsen, Peter Roos, Christina Vangsted-Hansen, Tua Vinther-Jensen,

**Odense (Odense University Hospital):** Annette Lolk, Marianne Lundsgaard, Lene Wermuth

# FINLAND

**Aland:** Christian Andersson, Clara Nyberg, Jimmy Sundblom

**Oulu (Dep. of Neurology):** Jaana Åman, Jaakko Ignatius, Mikko Kärppä

**Oulu (Dep. of Medical Genetics):** Aki Mustonen, Outi Kajula, Outi Jääskalainen, Jukka Moilanen

**Tampere (Terveystalo Healthcare Service Centre):** Maire Santala

**Turku-Suvituuli (Rehabilitation Centre Suvituuli):** Pia Eklund, Heli Hiivola, Hannele Hyppönen, Kirsti Martikainen, Katri Tuuha

**FRANCE**

**Angers (Centre de référence des maladies neurogénétique- CHU d’Angers):** Philippe Allain, Dominique Bonneau, Marie Bost, Bénédicte Gohier, Marie-Anne Guérid, Audrey Olivier, Julie Prouzet, Adriana Prundean, Clarisse Scherer-Gagou, Christophe Verny

**Bordeaux (Hôpital Pellegrin):** Blandine Babiloni, Déborah Bled, Sabrina Debruxelles, Charlotte Duché, Cyril Goizet, Laetitia Jameau, Danielle Lafoucrière, Umberto Spampinato

**Clermont-Ferrand (Hôpital Gabriel Montpied):** Julien Couttier, Bérengère Debilly, Christine Delaigue, Franck Durif, Véronique Germain, Perrine Legendre, Sylvie Loiseau, Miguel Ulla, Tiphaine Vidal

**Creteil (Hôpital Henri Mondor):** Anne-Catherine Bachoud-Lévi, Farideh Badei, Marie-Françoise Boissé, Lotfi Boudali, Laurent Cleret de Langavant, Laurie Lemoine, Graca Morgado, Katia Youssov

**Lille-Amiens:**

**Lille (CHRU Roger Salengro):** Agnès Annic, Recka Barthélémy, Christelle De Bruycker, Maryline Cabaret, Anne-Sophie Carette, Nicolas Carrière, Eric Decorte, Luc Defebvre, Marie Delliaux, Arnaud Delval, Alizé Depelchin, Alain Destee, Nelly Dewulf-Pasz , Thibaut Dondaine, Florence Dugauquier, Kathy Dujardin, Lucie Hopes, Pierre Krystkowiak, Marie-Hélène Lemaire, Sylvie Manouvrier, Eugénie Mutez, Mireille Peter, Lucie Plomhause, Bernard Sablonnière, Clémence Simonin, Céline Tard, Stéphanie Thibault-Tanchou, Isabelle Vuillaume

**Amiens (CHU Sud):** Marcellin Bellonet, Alexandra Benoit, Hassan Berrisoul, Stéphanie Blin, Françoise Courtin, Cécile Duru, Véronique Fasquel, Mélanie Flament, Olivier Godefroy, Pierre Krystkowiak, Béatrice Mantaux, Alicia Playe, Martine Roussel, Mélissa Tir, Béatrice Schüler, Sandrine Wannepain

**Marseille (Hôpital La Timone):** Jean-Philippe Azulay, Christelle Chabot, Marie Delfini, Alexandre Eusebio, Frédérique Fluchere, Christine Garreau, Hélène Grosjean, Aicha Guenam, Laura Mundler, Marielle Nowak Rolland Raseta

**Paris (Hôpital de la Pitié Salpêtrière):** Sandra Benaich, Alexis Brice, Sarah Boster, Perrine Charles, Alexandra Durr, Claire Ewenczyk, Hélène Francisque, Céline Jauffret, Damian Justo, Abdulrahman Kassar, Stephan Klebe, Fabien Lesne, Paolo Milani, Marie-Lorraine Monin, Tiffany Monnier, Emmanuel Roze, Alina Tataru, Maya Tchikviladzé

**Rouen (Hôpital Charles Nicolle):** Sandrine Bioux, Evangeline Bliaux, Carole Girard, Lucie Guyant-Maréchal, Didier Hannequin, Véronique Hannier, Séverine Jourdain, David Maltête, Dorothée Pouliquen

**Strasbourg (Hôpital Civil):** Mathieu Anheim, Nadia Barun, Ouhaid Lagha-Boukbiza, Nadine Longato, Christophe Marcel, Clélie Phillipps, Gabrielle Rudolf, Gisèle Steinmetz , Christine Tranchant , Caroline Wagner , Marie-Agathe Zimmermann

**Toulouse (Hôpital Purpan):** Leily Blondeau, Fabienne Calvas, Samia Cheriet, Helène Delabaere, Jean-François Demonet, Jérémie Pariente,  Michèle Pierre, Sandrine Rolland

**GERMANY**

**Aachen (Universitätsklinikum Aachen, Neurologische Klinik):** Christoph Michael Kosinski, Eva Milkereit, Daniela Probst, Kathrin Reetz, Christian Sass, Johannes Schiefer, Christiane Schlangen, Cornelius J. Werner

**Berlin (Universitätsmedizin Berlin, Klinik und Poliklinik für Neurologie):** Markus Beuth, Harald Gelderblom, Josef Priller, Harald Prüß, Eike Spruth, Silvia Thiel

**Bochum (Huntington-Zentrum (NRW) Bochum im St. Josef-Hospital):** Jürgen Andrich, Gisa Ellrichmann, Lennard Herrmann, Rainer Hoffmann, Barbara Kaminski, Peter Kraus, Carsten Saft, Christiane Stamm

**Dinslaken (Reha Zentrum in Dinslaken im Gesundheitszentrums Lang):** Herwig Lange, Robert Maiwald

**Dresden (Universitätsklinikum Carl Gustav Carus an der Technischen Universität Dresden, Klinik und Poliklinik für Neurologie):** Cecile Bosredon, Ulrike Hunger, Matthias Löhle, Antonia Maass, Christiana Ossig, Simone Schmidt, Alexander Storch, Annett Wolz, Martin Wolz

**Erlangen (Universitätsklinikum Erlangen, Molekulare Neurologie und Klinik für Neurologie):** Zacharias Kohl, Christina Kozay, Jasmin Ullah, Jürgen Winkler

#### Freiburg (Universitätsklinik Freiburg, Neurologie): Ulrike Bergmann, Regina Böringer, Philipp Capetian, Gerit Kammel, Johann Lambeck, Miriam Mächtel, Simone Meier, Michel Rijntjes, Birgit Zucker

**Hamburg (Universitätsklinikum Hamburg-Eppendorf, Klinik und Poliklinik für Neurologie):** Kai Boelmans, Christos Ganos, Ines Goerendt, Walburgis Heinicke, Ute Hidding, Jan Lewerenz, Alexander Münchau, Michael Orth, Jenny Schmalfeld, Lars Stubbe, Simone Zittel

**Hannover (Neurologische Klinik mit Klinischer Neurophysiologie, Medizinische Hochschule Hannover):** Gabriele Diercks, Dirk Dressler, Flverly Francis, Sabine Gayde-Stephan, Heike Gorzolla, Bianca Kramer, Rebecca Minschke, Christoph Schrader, Pawel Tacik

**Itzehoe (Schwerpunktpraxis Huntington, Neurologie und Psychiatrie):** Michael Ribbat+

**Marburg KPP (Klinik für Psychiatrie und Psychotherapie Marburg-Süd):** Bernhard Longinus

**München (Huntington-Ambulanz im Neuro-Kopfzentrum - Klinikum rechts der Isar der Neurologischen Klinik und Poliklinik der Technischen Universität München):** Antje Lüsebrink, Mark Mühlau, Alexander Peinemann, Michael Städtler, Adolf Weindl, Juliane Winkelmann, Cornelia Ziegler

**Münster (Universitätsklinikum Münster, Klinik und Poliklinik für Neurologie):** Natalie Bechtel, Heike Beckmann, Stefan Bohlen, Nicole Göpfert, Eva Hölzner, Herwig Lange, Ralf Reilmann, Stefanie Rohm, Silke Rumpf, Christian Sass, Sigrun Schepers, Nathalia Weber

**Taufkirchen (Isar-Amper-Klinikum - Klinik Taufkirchen (Vils)):** Michael Bachmeier, Matthias Dose, Nina Hofstetter, Ralf Marquard, Alzbeta Mühlbäck

**Ulm (Universitätsklinikum Ulm, Neurologie):** Katrin Barth, Andrea Buck, Julia Connemann, Daniel Ecker, Carolin Geitner, Christine Held, Andrea Kesse, Bernhard Landwehrmeyer, Franziska Lezius, Jan Lewerenz, Solveig Nepper, Anke Niess, Michael Orth, Ariane Schneider, Daniela Schwenk, Sigurd Süssmuth, Sonja Trautmann, Melanie Vogel, Patrick Weydt

**Würzburg (Universitätsklinikum Würzburg, Neurologie):** Stephan Klebe, Thomas Musacchio, Christine Leypold, Kerstin Nöth

**ITALY**

**Bari (Neurophysiopathology of Pain Unit, Basic Medical, Neuroscience and Sensory System Department, University of Bari):** Claudia Cormio, Olimpia Difruscolo, Giovanni Franco, Vittorio Sciruicchio, Claudia Serpino, Marina de Tommaso

**Bologna (DIBINEM - Alma Mater Studiorum - Università di Bologna, IRCCS Istituto delle Scienze Neurologiche di Bologna):** Giovanna Calandra-Buonaura, Sabina Capellari, Pietro Cortelli, Roberto Gallassi, Roberto Poda, Cesa Scaglione

**Cagliari (Movement Disorders Center, Department of Neurology, Institute of Neurology, University of Cagliari):** Michela Figorilli, Francesco Marrosu, Antonella Muroni, Valeria Piras, Melisa Vacca

**Florence (Department of Neuroscience, University of Florence** **& Careggi University Hospital*)*:** Elisabetta Bertini, Caterina Bartoli, Fernanda Fortunato, Elena Ghelli, Andrea Ginestroni, Claudia Mechi, Marco Paganini, Silvia Piacentini, Silvia Pradella, Anna Maria Romoli, Sandro Sorbi

**Genoa (Department of Neuroscience, Rehabilitation, Ophthalmology, Genetics, Maternal and Child Health, University of Genova):** Giovanni Abbruzzese, Monica Bandettini di Poggio, Giovanna Ferrandes, Paola Mandich, Roberta Marchese, Emilio Di Maria, Tiziano Tamburini

**Milan (SODS Genetica delle Malattie Neurodegenerative e Metaboliche & U.O. Neurologia, Fondazione IRCCS Istituto Neurologico Carlo Besta):** Alberto Albanese, Simona Castagliuolo, Anna Castaldo, Stefano Di Donato, Daniela Di Bella, Cinzia Gellera, Silvia Genitrini, Caterina Mariotti, Daniela Monza, Lorenzo Nanetti, Marta Panzeri, Dominga Paridi, Paola Soliveri, Francesca Spagnolo, Franco Taroni, Chiara Tomasello

**Naples (Department of Neurosciences and Reproductive and Odontostomatological Sciences, Federico II University of Naples):** Giuseppe De Michele, Luigi Di Maio, Carlo Rinaldi, Marco Massarelli, Silvio Peluso, Alessandro Roca, Cinzia Valeria Russo, Elena Salvatore, Pierpaolo Sorrentino, Tecla Tucci

**Pozzilli (IS) (IRCCS Neuromed):** Milena Cannella, Valentina Codella, Francesca De Gregorio, Annunziata De Nicola, Francesca Elifani, Tiziana Martino, Francesca Lovo, Irene Mazzante, Martina Petrollini, Maria Simonelli, Ferdinando Squitieri, Maurizio Vezza

**Rome (LIRH Foundation):** Barbara D'Alessio, Chiara Esposito, Irene Mazzante, Ferdinando Squitieri

**Rome (Department of Neurology, Università Cattolica del Sacro Cuore; Institute of Translational Pharmacology & Institute of Cognitive Sciences and Technologies, National Research Council of Italy):** Anna Rita Bentivoglio, Francesco Bove, Claudio Catalli, Raffaella Di Giacopo, Alfonso Fasano, Marina Frontali, Arianna Guidubaldi, Tamara Ialongo, Gioia Jacopini, Giovanna Loria, Anna Modoni, Martina Petracca, Carla Piano, Piccininni Chiara, Davide Quaranta, Silvia Romano, Francesco Soleti, Marcella Solito, Maria Spadaro, Flavia Torlizzi, Paola Zinzi

**Rome (Azienda Ospedaliera Sant'Andrea; Department of Neuroscience, Mental Health and Sensory Organs (NESMOS), Faculty of Medicine and Psychology, Sapienza University of Rome; Institute of Translational Pharmacology & Institute of Cognitive Sciences and Technologies, National Research Council of Italy):** Giulia Coarelli, Michela Ferraldeschi, Marina Frontali, Gioia Jacopini, Giovanni Ristori, Silvia Romano, Paola Zinzi

**NETHERLANDS**

**Enschede (Medisch Spectrum Twente):** Monique S.E. van Hout, Jeroen P.P. van Vugt, A. Marit de Weert, Marloes Verhoeven

**Groningen (Polikliniek Neurologie):** Meike Dekker, Jesper Klooster, Nico Leenders, Joost van Oostrom, Jesper Klooster, Berry Kremer

**Leiden (Leiden University Medical Centre (LUMC)):** Verena Baake, Simon J. A. van den Bogaard, Reineke Bos, Eve M. Dumas, Ellen P. ‘t Hart, Milou Jacobs, Anne Kampstra, Raymund A.C. Roos, Anne Schoonderbeek

**Maastricht:** Annelien Duits, Mayke Oosterloo, Mirella Waber

**NORWAY**

**Bergen (Haukeland University Hospital, Dept of Medical Genetics and Olaviken Psychiatric Hospital):** Ellen Økland Blinkenberg. (NKS Olaviken`s HD clinic): Erik Hauge, Hilde Tyvoll

**Oslo University Hospital (Dept. of Medical Genetics, Dept. of Neurology, Dept.of Neurorehabilitation):** Olaf Aaserud, Nils Olaf Aanonsen, Kathrine Bjørgo, Nancy Borgeød, Elisabeth Dramstad, Madeleine Fannemel, Jan C. Frich, Per F. Gørvell, Kathrine Haggag), Cecilie Haggag Johannessen, Arvid Heiberg, Lars Retterstøl, Oddveig Røsby, Jutta Rummel, Alma Sikiric, Bodil Stokke, Marleen van Walsem, Ragnhild Wehus

**Trondheim (St. Olavs Hospital):** Inga Bjørnevoll, Sigrid Botne Sando, Marte Gjøl Haug, Hanna Haugan Størseth, Vibeke Arntsen

**POLAND**

**Gdansk (St. Adalbert Hospital, Gdansk, Medical University of Gdansk, Neurological and Psychiatric Nursing Dpt.):** Artur Dziadkiewicz, Agnieszka Konkel, Ewa Narożańska, Malgorzata Nowak, Piotr Robowski, Emilia Sitek, Jaroslaw Slawek, Witold Soltan, Michal Szinwelski

**Katowice (Medical University of Silesia, Katowice):** Michał Arkuszewski, Magdalena Błaszczyk, Magdalena Boczarska-Jedynak, Ewelina Ciach-Wysocka, Agnieszka Gorzkowska, Barbara Jasińska-Myga, Aleksandra Kaczmarczyk, Gabriela Kłodowska – Duda, Grzegorz Opala, Monika Rudzińska, Daniel Stompel

**Krakow (Krakowska Akademia Neurologii):** Krzysztof Banaszkiewicz, Dorota Boćwińska, Kamila Bojakowska-Jaremek, Małgorzata Dec, Natalia Grabska, Malgorzata Krawczyk, Ewelina Kubowicz, Michalina Malec-Litwinowicz, Monika Rudzińska, Agata Stenwak, Andrzej Szczudlik, Elżbieta Szczygieł, Magdalena Wójcik, Anna Wasielewska

**Poznan (Poznan University of Medical Sciences, Poland):** Jacek Anioła Anna Bryl, Anna Ciesielska, Aneta Klimberg, Jerzy Marcinkowski, Husam Samara, Justyna Sempołowicz, Bartłomiej Wiśniewski, Daniel Zielonka

**Warsaw-MU (Medical University of Warsaw, Neurology):** Anna Gogol (formerly Kalbarczyk), Piotr Janik, Zygmunt Jamrozik, Anna Kaminska, Hubert Kwiecinski+, Natalia Szejko

**Warsaw-IPiN (Institute of Psychiatry and Neurology Dep. of Genetics, First Dep. of Neurology):** Jakub Antczak, Katarzyna Jachinska, Wioletta Krysa, Maryla Rakowicz, Przemyslaw Richter, Rafal Rola, Danuta Ryglewicz, Halina Sienkiewicz-Jarosz, Iwona Stępniak, Anna Sułek, Grzegorz Witkowski, Jacek Zaremba, Elzbieta Zdzienicka, Karolina Ziora-Jakutowicz

**PORTUGAL**

**Coimbra – (Hospital Universitário de Coimbra):** Cristina Januário, Filipa Júlio

**Lisbon-Central (Hospital dos Capuchos, Centro Hositalar Lisboa Central):** Manuel Almeida, Ana Calado, Margarida Dias, Joana Morgado, Cristina Semedo

**Lisbon-HSM (Hospital de Santa Maria, Clinical Pharmacology Unit, Instituto de Medicina Molecular):** Leonor Correia Guedes, Miguel Coelho, Joaquim J Ferreira, Andreia Magalhães, Tiago Mestre, Tiago Mendes, Dulce Neutel, Filipe Rodrigues, Anabela Valadas

**Lisbon-HFF (Hospital Fernando da Fonseca):** Cristina Costa, Helena Cardoso, Tiago Mendes, Mariana Santos

**Porto- HSJ (Hospital de São João):** Carlos Andrade, Andreia Costa, Carolina Garrett, Miguel Gago, Joana Guimarães, João Massano, Joana Meireles, Ana Monteiro

**RUSSIAN FEDERATION**

**Moscow – (Research Center of Neurology):** Sergey Illarioshkin, Sergey Klyushnikov, Olga Sidorova, Oleg Smirnov, Elizaveta Yudina, Yury Seliverstov

**SPAIN**

**Badajoz (Hospital Infanta Cristina):** Carmen Durán Herrera, Patrocinio Garcia Moreno

**Barcelona-Bellvitge (Hospital Universitari de Bellvitge):** Jordi Bas, Núria Busquets, Matilde Calopa, Serge Jaumà Classen, Nadia Rodríguez Dedichá

**Barcelona- Clínic i Provincial (Hospital Clínic i Provincial):** María Teresa Buongiorno, Andrés de la Cerda Santa María, Esteban Muñoz, Pilar Santacruz

**Barcelona-Hospital Mútua de Terrassa:** Miquel Aguilar Barbera, Ana Rojo Sebastián, Sonia Arribas Pardo, Dolors Badenes Guia, Noemi Calzado, Laura Casas Hernanz, Juan Pablo Tartari Díaz-Zorita, Judit López Catena, Pilar Quiléz Ferrer, Gemma Tome Carruesco

**Barcelona-Merced (Hospital Mare de Deu de La Merced):** Misericordia Floriach Robert, Cèlia Mareca Viladrich, Elvira Roca, Jesús Miguel Ruiz Idiago, Antonio Villa Riballo

**Barcelona-Santa Cruz y San Pablo (Hospital de la Santa Creu i Sant Pau):** Antonia Campolongo, Ramon Fernandez de Bobadilla, Jaime Kulisevsky Bojarsky, Saul Martinez-Horta, Javier Pagonabarraga, Jesus Perez Perez, Roser Ribosa, Carolina Villa

**Bilbao (Hospital de Cruces):** Maria Angeles Acera Gil, Koldo Berganzo Corrales, Juan Carlos Gomez Esteban, Amaia González, Beatriz Tijero Merino

**Burgos (Servicio de Neurología Hospital General Yagüe):** Esther Cubo, Cecilia Gil Polo, Natividad Mariscal

**Canarias (Hospital Insular de Gran Canaria):** Sandra Gutierrez Romero, José Matías Arbelo, Rocío Malo de Molina, Idaira Martín, Juan Manuel Periañez, Beatriz Udaeta

**Fuenlabrada (Hospital Universitario):** Fernando Alonso-Frech, María del Valle Loarte

**Granada (Hospital Universitario San Cecilio, Neurología):** Francisco Barrero, Blas Morales

**Madrid-BTCIEN (Fundación CIEN):** Belén Frades, Marina Ávila Villanueva, Maria Ascension Zea Sevilla

**Madrid-Clinico (Hospital Clínico Universitario San Carlos):** Fernando Alonso Frech, María del Mar Fenollar, Rocío García-Ramos García, Clara Villanueva

**Madrid RYC (Hospital Ramón y Cajal, Neurología):** Mónica Bascuñana, Marta Fatás Ventura, Juan García Caldentey, Guillermo García Ribas, Justo García de Yébenes, José Luis López–Sendón Moreno, Verónica Mañanes Barral, Patricia Trigo Cubillo

**Madrid FJD (Madrid-Fundación Jiménez Díaz):** Cici Feliz Feliz, Pedro José García Ruíz, Ana García, Juan García Caldentey, Rosa Guerrero López, Antonio Herranz Bárcenas, Asunción Martínez-Descals, Angel Martínez Pueyo, Veronica Puertas Martin, Noelia Rodríguez Martínez, María José Sainz Artiga, Vicenta Sánchez, Javier del Val Fernandez

**Murcia (Hospital Universitario Virgen de la Arrixaca):** Moreau María Dolores Alarcón, Carmen Antúnez Almagro, Esther Diéguez, Lorenza Fortuna, Salvadora Manzanares, Juan Marín Muñoz, María Martirio Antequera Torres, Fuensanta Noguera Perea, Laura Vivancos

**Oviedo (Hospital Central de Asturias):** Sonia González, Luis Menéndez Guisasola, Marta Para Prieto, René Ribacoba, Carlos Salvador, Pablo Sánchez Lozano

**Palma de Mallorca (Hospital Universitario Son Espases):** Juan García Caldentey, Inés Legarda Ramirez, Penelope Navas Arques, Monica Rodriguez Lopera, Barbara Vives Pastor

**Pamplona (Complejo Hospitalario de Navarra):** Itziar Gaston, Fermin Garcia-Amigot, Maria Dolores Martinez-Jaurrieta, Maria Antonia Ramos-Arroyo

**Sevilla (Hospital Universitario Virgen del Rocío):** Fátima Carrillo, María Teresa Cáceres Redondo, Pablo Mir, Laura Vargas González

**Sevilla (Hospital Virgen Macarena):** Fátima Damas Hermoso, José Manuel García Moreno, Carolina Mendez Lucena, Eva María Pacheco Cortegana, José Chacón Peña, Luis Redondo, Violeta Sánchez Sánchez

### Valencia (Hospital la Fe): Maria Bosca, Juan Andres Burguera, Francisco Castera Brugada Carmen Peiró Vilaplana, Pilar Solís, Begoña Jeweinat Figuerola, Paloma Millan Palanca

**SWEDEN**

**Göteborg (Sahlgrenska University Hospital**): Jan Wahlström+, Ulrika Høsterey-Ugander, Gunnel Fredlund, Radu Constantinescu, Kajsa Lewin, Liselotte Neleborn-Lingefjärd, Maria Berglund, Peter Berglund, Petra Linnsand

**Lunds University Hospital:** Åsa Petersén, Jan Reimer, Håkan Widner

**Stockholm Karolinska University Hospital:** Stanislav Benaminov, Elisabeth Björnsson, Daniel Merrick, Martin Paucar, Sven Pålhagen, Per Svenningsson, Tina Wallden

**Umeå (Umeå University Hospital):** Måns Berglund, Ghada Loutfi, Carina Olofsson, Eva-Lena Stattin, Laila Westman, Birgitta Wikström

**Uppsala University Hospital:** Camilla Ekwall, Marie-Lousie Göller, Valter Niemelä, Jimmy Sundblom

**SWITZERLAND**

**Bern:** Jean-Marc Burgunder, Jessica Koehli, Yanik Stebler (Swiss HD Zentrum), Alain Kaelin, Irene Romero, Michael Schüpbach, Sabine Weber Zaugg (Zentrum für Bewegungsstörungen, Neurologische Klinik und Poliklinik, Universität Bern)

**Lausanne:** Federica Esposito, Jean-Marc Good, Karin Paus, Francois Vingerhoets, Christian Wider

**U.K.**

**Aberdeen (NHS Grampian Clinical Genetics Centre & University of Aberdeen):** Lorna Downie, Roisin Jack, Kirsty Matheson, Zosia Miedzybrodzka, Daniela Rae, Sheila A Simpson, Fiona Summers, Alexandra Ure, Vivien Vaughan

**Barnstaple:** Timothy Harrower, Nathan Vernon

**Birmingham (The Barberry Centre, Dept of Psychiatry):** Shahbana Akhtar, Jenny Crooks, Adrienne Curtis, Jenny de Souza (Keylock), John Piedad, Hugh Rickards, Jan Wright

**Bristol (North Bristol NHs Trust, Southmead hospital):** Elizabeth Coulthard, Louise Gethin, Beverley Hayward, Kasia Sieradzan, Abigail Wright

**Cambridge (Cambridge Centre for Brain Repair, Forvie Site**): Roger A. Barker, Deidre O’Keefe, Anna Gerrtiz (nee Di Pietro), Kate Fisher, Anna Goodman, Susan Hill, Sarah Mason, Rachel Swain, Natalie Valle Guzman

**Cardiff (Schools of Medicine and Biosciences, Cardiff University):** Monica Busse, Cynthia Butcher, Stephen Dunnett, Catherine Clenaghan, Ruth Fullam, Sarah Hunt, Lesley Jones, Una Jones, Hanan Khalil, Sara Minster,  Michael Owen, Kathleen Price, Jenny Townhill,  Anne Rosser

**Edinburgh (SE Scotland Genetic Service, Western General Hospital):** Maureen Edwards, Carrie Ho (Scottish Huntington´s Association), Marie McGill, Mary Porteous, Pauline Pearson

**Exeter (Department of Neurology Royal Devon and Exeter Foundation Trust Hospital):** Timothy Harrower, Sarah Irvine

**Fife (Scottish Huntington's Association Whyteman's Brae Hospital):** Peter Brockie, Jillian Foster, Nicola Johns, Sue McKenzie, Jean Rothery, Gareth Thomas, Shona Yates

**Forth Valley (Neurology Department, Forth Valley Royal Hospital):** Christian Neumann, Kirsten Patterson, David Thomson

**Glasgow (Glasgow HD Management Clinic, Southern General Hospital)**: Catherine Deith, Jane Ireland, Stuart Ritchie

**Gloucester (Department of Neurology Gloucestershire Royal Hospital):** Pauline Brown, Liz Burrows, Amy Fletcher, Alison Harding, Fiona Laver, Mark Silva, Aileen Thomson

**Hull (Castle Hill Hospital):** Carol Chu, Carole Evans, Deena Gallentree, Stephanie Hamer, Alison Kraus, Ivana Markova, Ashok Raman

**Launceston (Millaton Court):** Alyson Andrew, Julie Frost, Rupert Noad

**Leeds (Chapel Allerton Hospital, Department of Clinical Genetics):** Jeremy Cosgrove, Deena Gallantree, Stephanie Hamer, Emma Hobson, Stuart Jamieson , Alison Kraus, Mandy Longthorpe, Ivana Markova, Hannah Musgrave, Caroline Peacy, Ashok Raman, Liz Rowett, Jean Toscano, Sue Wild, Pam Yardumian

**Leicester (Leicestershire Partnership Trust, Mill Lodge):** Carole Clayton, Heather Dipple, Dawn Freire-Patino, Caroline Hallam, Julia Middleton

**Liverpool (Walton Centre for Neurology and Neurosurgery):** Sundus Alusi, Rhys Davies, Kevin Foy, Emily Gerrans, Louise Pate

**London (St. Georges-Hospital):** Uruj Anjum, Jan Coebergh, Charlotte Eddy, Nayana Lahiri, Meriel McEntagart, Michael Patton, Maria Peterson, Sarah Rose

**London (Guy's Hospital):** Thomasin Andrews, Andrew Dougherty, Charlotte Golding, Fred Kavalier, Hana Laing, Alison Lashwood, Dene Robertson, Deborah Ruddy, Alastair Santhouse, Anna Whaite

**London (The National Hospital for Neurology and Neurosurgery):** Thomasin Andrews, Stefanie Brown, Stefania Bruno, Elvina Chu, Karen Doherty, Charlotte Golding, Salman Haider, Davina Hensman, Nayana Lahiri, Monica Lewis, Marianne Novak, Aakta Patel, Nicola Robertson, Elisabeth Rosser, Sarah Tabrizi, Rachel Taylor, Thomas Warner, Edward Wild

**Manchester (Genetic Medicine, University of Manchester, Manchester Academic Health Sciences Centre and Central Manchester University Hospitals NHS Foundation Trust):** Natalie Arran, Judith Bek, Jenny Callaghan, David Craufurd, Ruth Fullam, Marianne Hare, Liz Howard, Susan Huson, Liz Johnson, Mary Jones, Ashok Krishnamoorthy, Helen Murphy, Emma Oughton, Lucy Partington-Jones, Dawn Rogers, Andrea Sollom, Julie Snowden, Cheryl Stopford, Jennifer Thompson, Iris Trender-Gerhard, Nichola Verstraelen (formerly Ritchie), Leann Westmoreland

**Newcastle-upon-Tyne (Centre for Life, Institute of Medical Genetics):** Ginette Cass, Lynn Davidson, Jill Davison, Neil Fullerton, Katrina Holmes, Suresh Komati, Sharon McDonnell, Zeid Mohammed, Karen Morgan, Lois Savage, Baldev Singh, Josh Wood

**Northampton (St Andrew’s Healthcare):** Elvina Chu, Caroline Knight, Mari O’Neill, Debasish Das Purkayastha

**Oxford (Oxford University Hospitals NHS Trust, Dept. of Neurosciences, University of Oxford):** Andrea H Nemeth, Gill Siuda, Ruth Valentine, Kathryn Dixon, Richard Armstrong

**Plymouth (Plymouth Huntington Disease Service, Mount Gould Hospital):** David Harrison, Max Hughes, Sandra Large, John O Donovan, Amy Palmer, Andrew Parkinson, Beverley Soltysiak, Leanne Timings, Josh Williams

**Poole (Brain Injury Service, Poole Hospital):** John Burn, Rebecca Weekes, Janet Craven, Wendy Bailey, Caroline Coleman, Diane Haig-Brown, Steve Simpson

**Preston (Neurology Department, Preston Royal Hospital):** Marianne Hare, Tahir Majeed, Nicola Verstraelen (Ritchie)

**Reading (Royal Berkshire Hospital):** Richard Armstrong, Kathryn Dixon, Wendy Barrett, Aileen Ho

**Sheffield (The Royal Hallamshire Hospital– Sheffield Children’s Hospital):** Oliver Bandmann, Alyson Bradbury, Helen Fairtlough, Kay Fillingham, Isabella Foustanos, Paul Gill, Mbombe Kazoka, Kirsty O’Donovan, Louise Nevitt, Nadia Peppa, Oliver Quarrell, Cat Taylor, Katherine Tidswell, Kirsty O’Donovan

**Southampton (Southampton General Hospital):** Christopher Kipps, Lesley MacKinnon, Veena Agarwal, Elaine Hayward, Kerry Gunner, Kayla Harris, Mary Anderson, Melanie Heywood, Liane Keys, Sarah Smalley

**Swindon (Victoria Centre, Great Western Hospital):** Lesley Gowers, Kingsley Powell, Pamela Bethwaite, Rachel Edwards, Kathleen Fuller, Michelle Phillips

**EHDN’s associate site in Singapore:**

**National Neuroscience Institute Singapore:** Louis Tan, Jean-Marc Burgunder, Puay Ngoh Lau, Emmanuel Pica
